# PASCAL mitral valve repair system versus MitraClip: comparison of transcatheter edge-to-edge strategies in complex primary mitral regurgitation

**DOI:** 10.1007/s00392-021-01845-8

**Published:** 2021-04-10

**Authors:** Muhammed Gerçek, Fabian Roder, Tanja K. Rudolph, Vera Fortmeier, Armin Zittermann, Volker Rudolph, Kai P. Friedrichs

**Affiliations:** 1 Clinic for General and Interventional Cardiology/Angiology, Herz- Und Diabeteszentrum NRW, Ruhr-Universität Bochum, Georgstraße 11, 32545 Bad Oeynhausen, Germany; 2 Clinic for Thoracic and Cardiovascular Surgery, Herz- Und Diabeteszentrum NRW, Ruhr-Universität Bochum, Bad Oeynhausen, Germany

**Keywords:** Primary mitral regurgitation; MitraClip, PASCAL, Transcatheter therapy

## Abstract

**Background:**

The PASCAL system is a novel device for edge-to-edge treatment of mitral regurgitation (MR). The aim of this study was to compare the safety and efficacy of the PASCAL to the MitraClip system in a highly selected group of patients with complex primary mitral regurgitation (PMR) defined as effective regurgitant orifice area (MR-EROA) ≥ 0.40 cm^2^, large flail gap (≥ 5 mm) or width (≥ 7 mm) or Barlow’s disease.

**Methods:**

38 patients with complex PMR undergoing mitral intervention using PASCAL (*n* = 22) or MitraClip (*n* = 16) were enrolled. Primary efficacy endpoints were procedural success and degree of residual MR at discharge. The rate of major adverse events (MAE) according to the Mitral Valve Academic Consortium (MVARC) criteria was chosen as the primary safety endpoint.

**Results:**

Patient collectives did not differ relevantly regarding pertinent baseline parameters. Patients` median age was 83.0 [77.5–85.3] years (PASCAL) and 82.5 [76.5–86.5] years (MitraClip). MR-EROA at baseline was 0.70 [0.68–0.83] cm^2^ (PASCAL) and 0.70 [0.50–0.90] cm^2^ (MitraClip), respectively. 3D-echocardiographic morphometry of the mitral valve apparatus revealed no relevant differences between groups. Procedural success was achieved in 95.5% (PASCAL) and 87.5% (MitraClip), respectively. In 86.4% of the patients a residual MR grade ≤ 1 + was achieved with PASCAL whereas reduction to MR grade ≤ 1 + with MitraClip was achieved in 62.5%. Neither procedure time number of implanted devices, nor transmitral gradient differed significantly. No periprocedural MAE according to MVARC occured.

**Conclusion:**

In this highly selected patient group with complex PMR both systems exhibited equal procedural safety. MitraClip and PASCAL reduced qualitative and semi-quantitative parameters of MR to an at least comparable extent.

**Graphic abstract:**

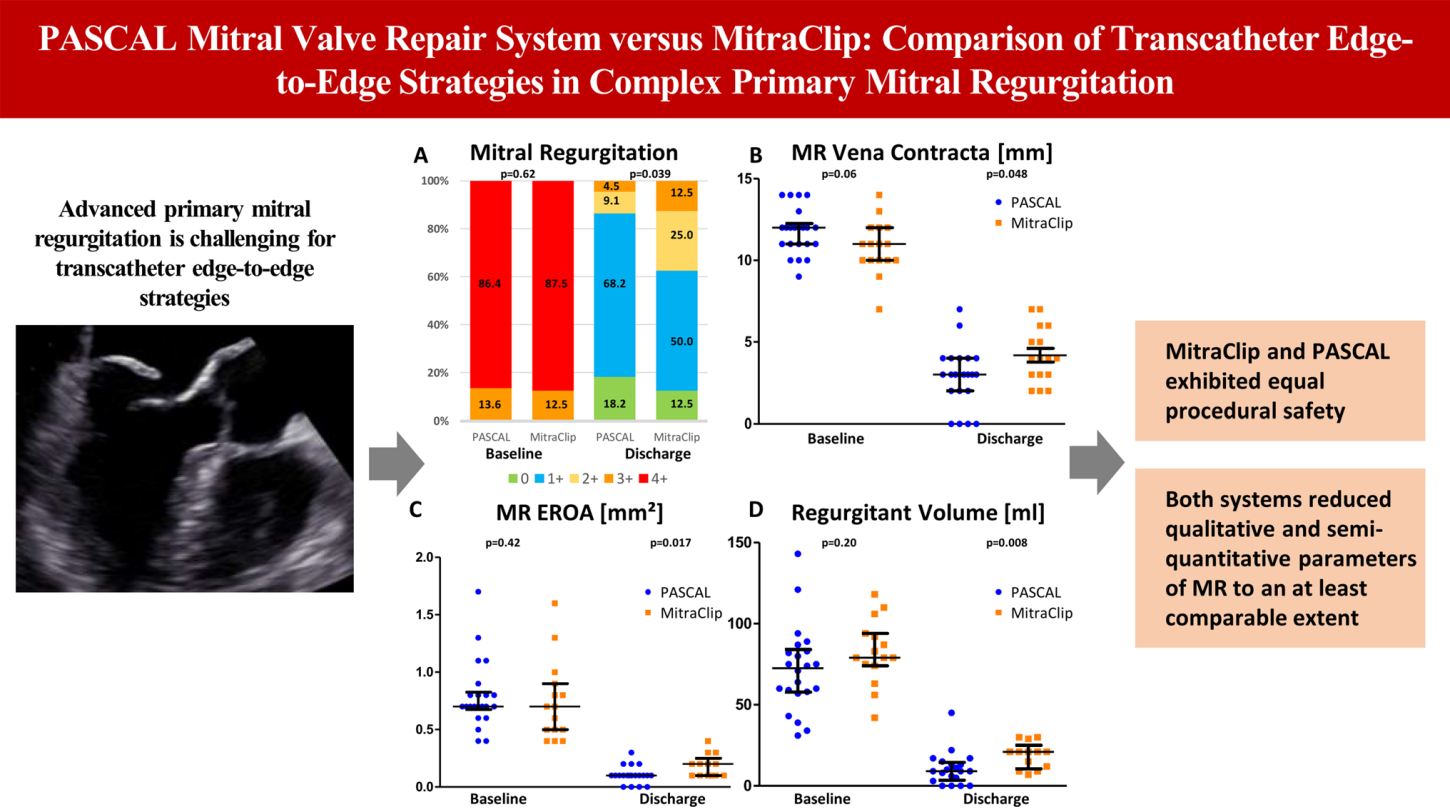

**Supplementary Information:**

The online version contains supplementary material available at 10.1007/s00392-021-01845-8.

## Introduction

The EVEREST II trial showed that edge-to-edge transcatheter mitral valve repair (TMVR) with the MitraClip system reduces mitral regurgitation less effectively than mitral valve surgery in a patient cohort with low surgical risk and a predominance of primary mitral regurgitation (PMR) [1]. The trial revealed that, while mortality and symptomatic improvement were similar after a 5-year follow-up, TMVR was associated with higher rates of residual/recurrent MR and mitral valve reinterventions [1]. Based on these findings surgery currently is considered the gold standard for MV repair in PMR in the absence of an increased perioperative risk and the advance of transcatheter mitral therapies will be critically dependent on achieving optimal procedural results even in patients with complex pathologies [2]. In this context, it is of interest that recently reported data from the EXPAND registry demonstrate a more pronounced effectiveness of the MitraClip system with a reduction of MR grade to ≤ 1 + in 94.5% in PMR patients [3].

With the PASCAL system a second device for TMVR has now become available, which offers distinctive features including a central spacer, broader paddles made of pliable nitinol and for the first time the possibility of independent leaflet grasping, which—in theory—provide technical advantages over the Gen3-MitraClip system [4]. A real-world study with over 300 patients recently accepted for publication indeed confirms promising results with high procedural success rates and safety for the PASCAL device while also achieving a similar effectiveness in a cohort of patients of whom one third presented with PMR [5]. This study directly compares the novel PASCAL device to the well-established MitraClip system for the first time particularly focusing on patients with complex and extensive degenerative mitral regurgitation.

## Methods

All consecutive patients with MR admitted for interventional treatment evaluation between August 2018 and April 2020 were retrospectively evaluated. 38 patients with complex PMR (defined as mitral regurgitation effective regurgitant orifice area (MR-EROA) ≥ 0.40 cm^2^ or large flail gap (≥ 5 mm) or width (≥ 7 mm) or Barlow`s disease) were included in the analysis. Therapeutic strategy (surgical or transcatheter) was chosen after heart team discussion for each case individually. The decision for the actual device used (MitraClip or PASCAL) was made at the discretion of the interventionalist. All interventions were performed by three interventional cardiologists who have each performed at least 300 TMVR procedures. The study was approved by the local Ethics Committee of the Ruhr University of Bochum and carried out in accordance with the Declaration of Helsinki.

### Echocardiographic assessment and implantation procedure

All patients underwent transthoracic and transesophageal echocardiography (Vivid E95, General Electric Healthcare, USA IL) before intervention. The images were stored digitally in a rapid mass storage system. Echocardiographic examinations were performed following the recommendations of the American Society of Echocardiography. MR severity was graded using transthoracic echocardiography at baseline and discharge using an multiparametric approach with the grade none/trace (1 +), mild to moderate (2 +), moderate to severe (3 +) and severe (4+) [6]. Detailed morphological analysis of the mitral valve and annulus were performed based on transoesophageal echocardiographic images obtained directly prior to the procedure using the mitral valve quantification (MVQ) analysis tool (EchoPac Version 203 (Revision 66.0) Vivid E95, General Electric Healthcare, USA IL) according to the recommendations of the manufacturer. All echocardiographic examinations were carried out by two independent investigators and checked for their validity including the MVQ analysis. Interobserver reliability of echocardiographic assessments is expressed as correlation coefficients with lower and upper bounds [7].

The procedures have been described in detail elsewhere [1, 4]. Both procedures amount to an edge-to-edge-approach for valve-repair. The main differences include the device properties (MitraClip: cobalt-chromium; PASCAL: nitinol), the central spacer and broader paddles of the PASCAL device. In addition, independent leaflet grasping is possible with the PASCAL device.

### Efficacy and safety endpoint

Efficacy and safety endpoints were defined based on the Mitral Valve Academic Research Consortium (MVARC) Criteria [8]. The efficacy endpoint was defined as reduction at least to moderate MR (2 +) at the end of the procedure, successful access and retrieval of the device delivery system with adequate deployment of the device after correct positioning and absence of procedural mortality, device-related reintervention and emergency surgery at discharge (procedural success). The safety endpoint was defined as the absence of procedural mortality, device-related reintervention, major adverse events within 30 days including all-cause mortality, stroke, acute kidney injury, severe bleeding (major, extensive, life-threatening, or fatal bleeding) and no need for device-related emergency surgery.

### Statistical analysis

Statistical analysis was performed using the SPSS-Software (Version 22, IBM Corporation, Armonk, NY, USA). Continuous variables are reported as median and interquartile range (IQR). Categorical variables are presented as frequencies and percentages. Student’s *t* test for unpaired and paired parametric samples or their analogues for nonparametric samples (Mann–Whitney and Wilcoxon signed rank) or the chi-squared test were performed for group comparisons, where appropriate. We generated Kaplan–Meier estimates to investigate the association of study group with survival or hospitalization probability during follow-up as a function of time after intervention. The log-rank test was used to test for outcome differences between groups. Because of non-randomized group assignment, we also performed a matched propensity score (PS) analysis in addition to the unmatched analysis. The PS was estimated by multivariable logistic regression. In the regression model, study group was the dependent variable. Regurgitant volume, diabetes mellitus, chronic obstructive pulmonary disease, coronary artery disease, peripheral artery disease, pre-operative stroke, and ICD/CRT implantation were selected as independent variables for PS matching. Matching was performed using a 1:1 ratio with the logit-transformed PS. For this, an optimal-matching algorithm with a caliper width of 0.2 standard deviation from the linear predictor was used. Standardized differences between groups were calculated as recommended by Yang and Dalton [9]. A *p*-value < 0.05 was considered significant. Interobserver reliability (IOR) was assessed using the interclass correlation for parametric values and the Cohen’s kappa coefficient for non-parametric values. IOR ≥ 0.7 was considered acceptable.

## Results

### Patients characteristics and baseline echocardiographic parameters

38 patients with primary mitral regurgitation were treated with percutaneous edge-to-edge mitral valve repair between August 2018 and April 2020. 22 patients received a PASCAL device and 16 patients underwent a MitraClip intervention (12 cases treated with the XTR-device, 2 cases treated with the NTR-device and 2 cases treated with a combination of both MitraClip devices). Detailed patient information is given in Table 1. Patients did not differ relevantly with regard to age, sex or comorbidities. Median age of the patients was 83.0 [77.5–85.3] years in the PASCAL group (PA) and 82.5 [76.5–86.5] years in the MitraClip group (MC). 40.9% (PA)/ 43.8% (MC) were female. Patients were considered at least at moderate surgical risk with a median EuroScore II of 4.0 [2.6–7.3] % (PA) and 3.8 [2.6–6.3] % (MC), respectively.

**Table 1:** Baseline characteristics of the study groups. Values are given as median [IQR] or percentages (*n*)

Characteristics	PASCAL (*n* = 22)	MitraClip (*n* = 16)	Standardized difference	*p* value
Age	83.0 [77.5–85.3]	82.5 [76.5–86.5]	0.17	0.80
Female	40.9% (9)	43.8% (7)	– 0.06	0.86
Body mass index kg/m^2^	25.8 [23.6–28.4]	24.5 [22.7–27.8]	0.36	0.43
STS-Score (%)	2.5 [1.8–5.2]	2.1 [1.0–4.0]	0.31	0.36
EuroScore II (%)	4.0 [2.6–7.3]	3.8 [2.6–6.3]	– 0.16	0.95
Atrial fibrillation	68.2% (15)	62.5% (10)	0.12	0.74
Diabetes mellitus	18.2% (4)	6.3% (1)	0.37	0.37
Chronic obstructive pulmonary disease	18.2% (4)	37.5% (6)	– 0.44	0.27
Coronary artery disease	36.4% (8)	50.0% (8)	– 0.28	0.51
History of myocardial infarction	4.5% (1)	12.5% (2)	– 0.29	0.56
History of cardiac surgery	22.7% (5)	12.5% (2)	0.27	0.68
Extracardiac arteriopathy	18.2% (4)	18.8% (3)	– 0.01	0.96
Stroke	13.6% (3)	25.0% (4)	– 0.29	0.43
Dialysis	4.5% (1)	0.0% (0)	0.31	0.39
ICD/CRT-Device	9.1% (2)	25.0% (4)	– 0.43	0.28
NTpro-BNP [pg/ml]	2410 [1105–5190] (*n* = 19)^a^	3000 [1502–4585] (*n* = 9)	0.26	0.65

^a^Dialysis patient was excluded from the analysis

68.2% of the patients in the PA group and 62.5% in the MC group presented with atrial fibrillation and 22.7% (PA; 12.5% MC) had a history of cardiac surgery. All patients were in New York Heart Association (NYHA) functional class III or IV. Some of the patient characteristics showed a moderate effect size of the group differences. Thus, PASCAL patients tended to have a slightly higher surgical risk based on STS, had a statistically non-significant higher prevalence of diabetes and dialysis; however, lower prevalences of chronic obstructive pulmonary disease and ICD/CRT devices.

Baseline echocardiographic parameters and values from MVQ analysis are presented in Table 2. More detailed parameters are given in Supplementary Tables 1 and 2. All patients suffered from moderate to severe (3 +) or severe (4 +) complex primary mitral regurgitation as defined above. Leaflet flail caused mitral regurgitation in 72.7% of the cases (PA; 75% in MC), while in 27.3% in PA (25% in MC) leaflet prolapse was the underlying pathology.

**Table 2:** Baseline echocardiographic parameters: values are given as median [IQR] or percentages (*n*)

Echocardiographic Parameters	PASCAL	MitraClip	Standardized difference	*p* value
Mitral regurgitation degree [I–IV]	III: 13.6% (3) IV: 86.4 (19) IOR 0.776	III: 12.5% (2) IV: 87.5% (14) IOR 0.636	< – 0.01	0.62
MV pathology	Prolapse: 27.3% (6) Flail: 72.7% (16)	Prolapse: 25.0% (4) Flail: 75.0% (12)	– 0.02	0.88
Vena contracta [mm]	12 [11, 12] IOR 0.727 [0.343–0.887] (*n* = 22)	11 [10–12] IOR 0.765 [0.328–0.918] (*n* = 16)	0.63	0.06
Effective regurgitant orifice area [cm^2^]	0.70 [0.68–0.83] IOR 0.858 [0.651–0943] (*n* = 22)	0.70 [0.50–0.90] IOR 0.955 [0.852–0.986] (*n* = 15)	0.16	0.42
Regurgitant volume [ml]	73 [58–84] IOR 0.807 [0.525–0.922] (*n* = 22)	79 [74–94] IOR 0.961 [0.543–0.957] (*n* = 15)	– 0.45	0.20
Proximal isovelocity surface area (PISA) radius adjusted to Nyquist limit 30–40 cm/s [mm] baseline	11 [9–13] IOR 0.951 [0.883–0.980] (*n* = 22)	11 [10–13] IOR 0.915 [0.758–0.970] (*n* = 16)	– 0.07	0.12
Flail gap [mm]	3.0 [1.0–5.0] (*n* = 16)	2.5 [0.5–6.0] (*n* = 12)	– 0.21	0.52
Flail width [mm]	9.0 [7.0–11.0] (*n* = 16)	9.5 [8.0–12.5] (*n* = 12)	– 0.27	0.38
Anterior billowing [mm]	1.0 [0–6.0] (*n* = 5)	0.5 [0–1.0] (*n* = 7)	0.24	0.19
Posterior billowing [mm]	2.0 [1.0–5.0] (*n* = 15)	2.0 [1.0–4.0] (*n* = 14)	– 0.20	0.91
Transmitral antegrade gradient [mmHg]	2.0 [1.0–3.0] (*n* = 22)	2.5 [2.0–3.0] (*n* = 16)	– 0.40	0.18
Mitral valve orifice area [cm^2^]	4.3 [3.9–5.7] (*n* = 22)	4.3 [3.9–6.3] (*n* = 16)	– 0.13	0.71
Annulus area 3D [cm^2^]	11.7 [10.7–13.1] (*n* = 22)	10.9 [9.0–15.3] (*n* = 16)	– 0.01	0.97
Annulus perimeter [cm]	12.5 [11.7–13.3] (*n* = 22)	12.1 [10.9–14.1] (*n* = 16)	0.06	0.86
A–P diameter [cm]	3.4 [3.0–3.7] (*n* = 22)	3.1 [3.0–4.0] (*n* = 16)	– 0.08	0.80
AL–PM diameter [cm]	3.9 [3.6–4.3] (*n* = 22)	3.7 [3.1–4.4] (*n* = 16)	0.33	0.29
Anterior leaflet length [cm]	2.1 [1.9–2.4] (*n* = 22)	2.3 [2.0–2.6] (*n* = 16)	– 0.37	0.26
Posterior leaflet length [cm]	1.8 [1.5–2.0] (*n* = 22)	1.4 [1.2–2.3] (*n* = 16)	0.12	0.71
Anterior leaflet area [cm^2^]	5.5 [5.1–6.3] (*n* = 22)	5.5 [4.2–7.5] (*n* = 16)	– 0.17	0.59
Posterior leaflet area [cm^2^]	7.2 [6.1–8.6] (*n* = 22)	7.8 [4.8–9.7] (*n* = 16)	– 0.09	0.77
Commissural diameter [cm]	3.7 [3.4–4.1] (*n* = 22)	3.6 [3.0–4.0] (*n* = 16)	0.40	0.22
Tricuspid regurgitation [0-V]	0: 4.5% (1) I: 59.1% (13) II: 27.3% (6) III: 9.1% (2) (*n* = 22)	I: 50.0% (8) II: 50.0% (8) (*n* = 16)	– 0.11	0.31
Estimated systolic pulmonary arterial pressure [mmHg]	43 [30–56] (*n* = 21)	49 [42–68] (*n* = 15)	– 0.47	0.18

**Fig. 1:**
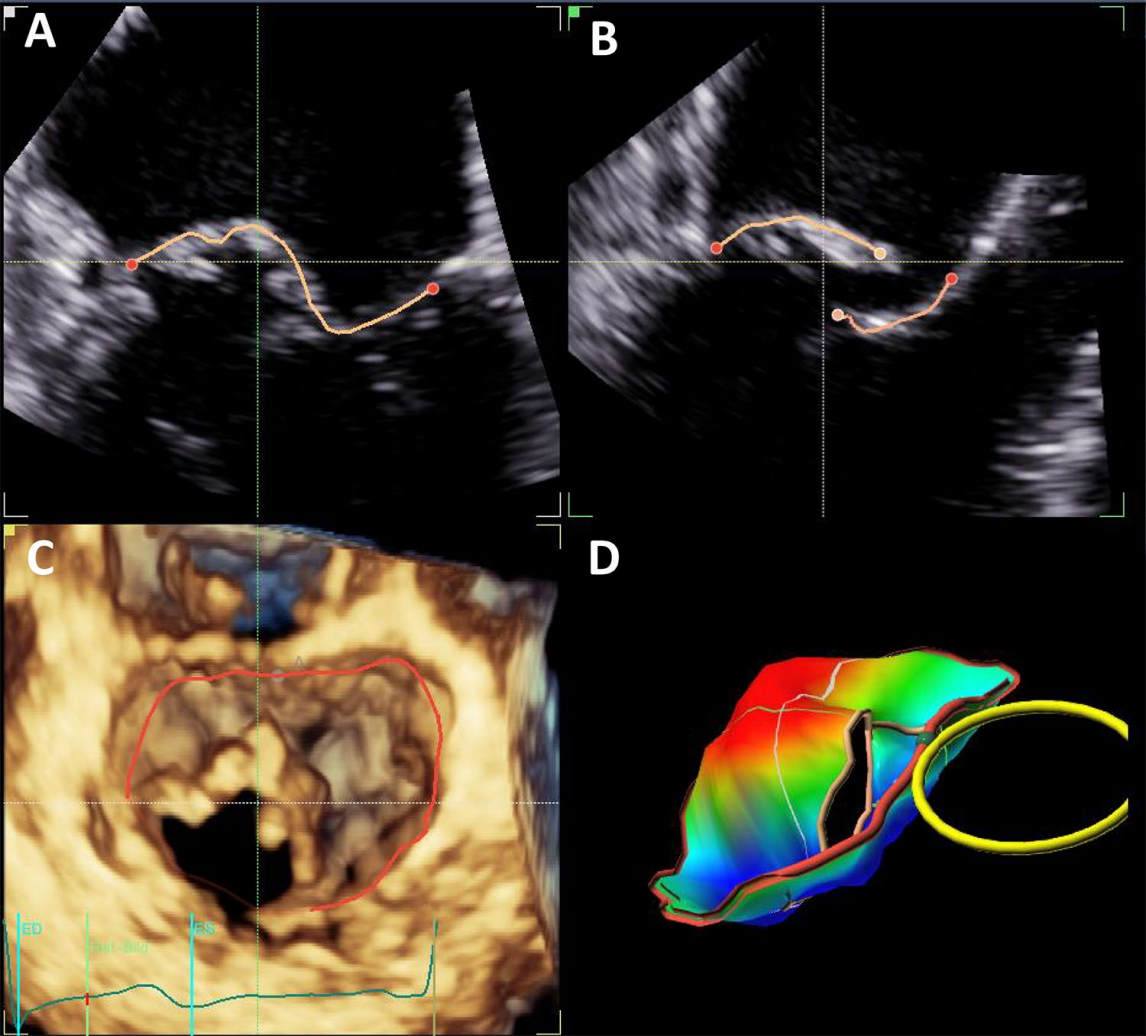
4D-echocardiography with tissue movement cartography. Exemplary illustration of the Mitral Valve Quantification (MVQ) Analysis of the morphological and annulus characteristics of the mitral valve (**a**–**d**)

Mean vena contracta (VC), effective regurgitant orifice area (EROA) and regurgitant volume did not differ significantly. The mean estimated systolic pulmonary arterial pressure (PAPsys) was elevated in both groups. In cases of mitral valve flail, median flail gap was 3 [0.5–6] mm (PA; 2.5 [0.5–6] mm MC) and median flail width 9 [7–11] mm (PA; 9.5 [8–12.5] mm MC). MVQ-analysis revealed similar morphological features of the mitral valve apparatus (Fig. 1, Table 2). Detailed mitral valve and leaflet parameters are shown in Supplementary Table 2.

### Periprocedural and echocardiographic results

Periprocedural outcomes are summarized in Table 3. On average, 2 [1, 2] devices were implanted in the PA group and 2 [1–3] devices in the MC group (*p* = 0.59). The time to procedure completion was 106.0 [81.3–123.3] min (PA) and 93 [71–132] min (MC; *p* = 0.930) with a fluoroscopy time of 7.8 [5.2–10.5] min (PA) and 9.5 [7.2–14.2] min (MC; *p* = 0.930), respectively (*p* = 0.88). Procedural success was achieved in 95.5% (PA) and 87.5% (MC), respectively. None of the patients in both groups suffered from a periprocedural major adverse event according to the MVARC criteria [8].

**Table 3:** Peri- and postprocedural results of transcatheter mitral valve repair with the PASCAL and MitraClip system. Values are given as median [IQR] or percentages (*n*)

Peri- and postprocedural parameters	PASCAL	MitraClip	*p* value
Number of implanted devices	2 [1-2] (n = 22)	2 [1-2] (*n* = 16)	0.59
Procedure time [min]	106.0 [81.3–123.3] (n = 22)	93.0 [71.0–132.0] (*n* = 16)	0.93
Fluoroscopy time [min]	7.8 [5.2–10.5] (n = 22)	9.5 [7.2–14.2] (*n* = 16)	0.10
Radiation dose area product [cGy*cm^2^]	429.1 [215.9–726.3] (*n* = 22)	367.5 [283.9–835.4] (*n* = 16)	0.88
Mitral regurgitation degree [0–IV]	**0:** 18.2% (4) **I:** 68.2% (15) **II:** 9.1% (2) **III:** 4.5% (1) IOR 0.817	**0:** 12.5% (2) **I:** 50.0% (8) **II:** 25.0% (4) **III:** 12.5% (2) IOR 0.909	**0.039**
Effective regurgitant orifice area [cm^2^]	0.10 [0.10–0.10] IOR 0.902 [0.794–0.958] (*n* = 20)	0.20 [0.10–0.25] IOR 0.895 [0.721–0.967] (*n* = 13)	**0.017**
Regurgitant volume [ml]	9 [4–15] IOR 0.914 [0.821–0.962] (*n* = 20)	21 [11–25] IOR 0.939 [0.850–0.979] (*n* = 13)	**0.008**
Vena contracta [mm]	3 [2–4] IOR 0.896 [0.784–0.954] (*n* = 21)	4 [3–6] IOR 0.942 [0.852–0.981] (*n* = 13)	**0.048**
PISA radius adjusted to Nyquist limit 30–40 cm/s [mm]	3 [3–4] IOR 0.953 [0.865–0.984] (*n* = 22)	4 [3–5] IOR 0.983 [0.958–0.993] (*n* = 16)	**0.043**
Transmitral gradient [mmHg]	3.0 [2.8–5.0] IOR 0.928 [0.854–0.968] (*n* = 22)	3.0 [2.0–4.8] IOR 0.978 [0947–0.992] (*n* = 16)	0.61
Estimated systolic pulmonary arterial pressure [mmHg]	34 [27–42] (*n* = 19)	30 [23–33] (*n* = 15)	0.23
Δ Vena contracta (Baseline-Discharge) [mm]	9 [7–11] (*n* = 22)	7 [6–8] (*n* = 13)	**0.003**
Δ Effective regurgitant orifice area (Baseline-Discharge) [cm^2^]	0.60 [0.42–0.78] (*n* = 20)	0.50 [0.30–0.70] (*n* = 13)	0.64
Δ Regurgitant Volume (Baseline-Discharge) [ml]	59 [44–76] (*n* = 20)	67 [51–76] (*n* = 13)	0.45
Δ Pisa radius adjusted to Nyquist limit 30–40 cm/s (Baseline-discharge) [mm]	7 [6–10] (*n* = 22)	7 [5–8] (*n* = 16)	0.24

Bold values indicate significant *p* values < 0.05

**Fig. 2:**
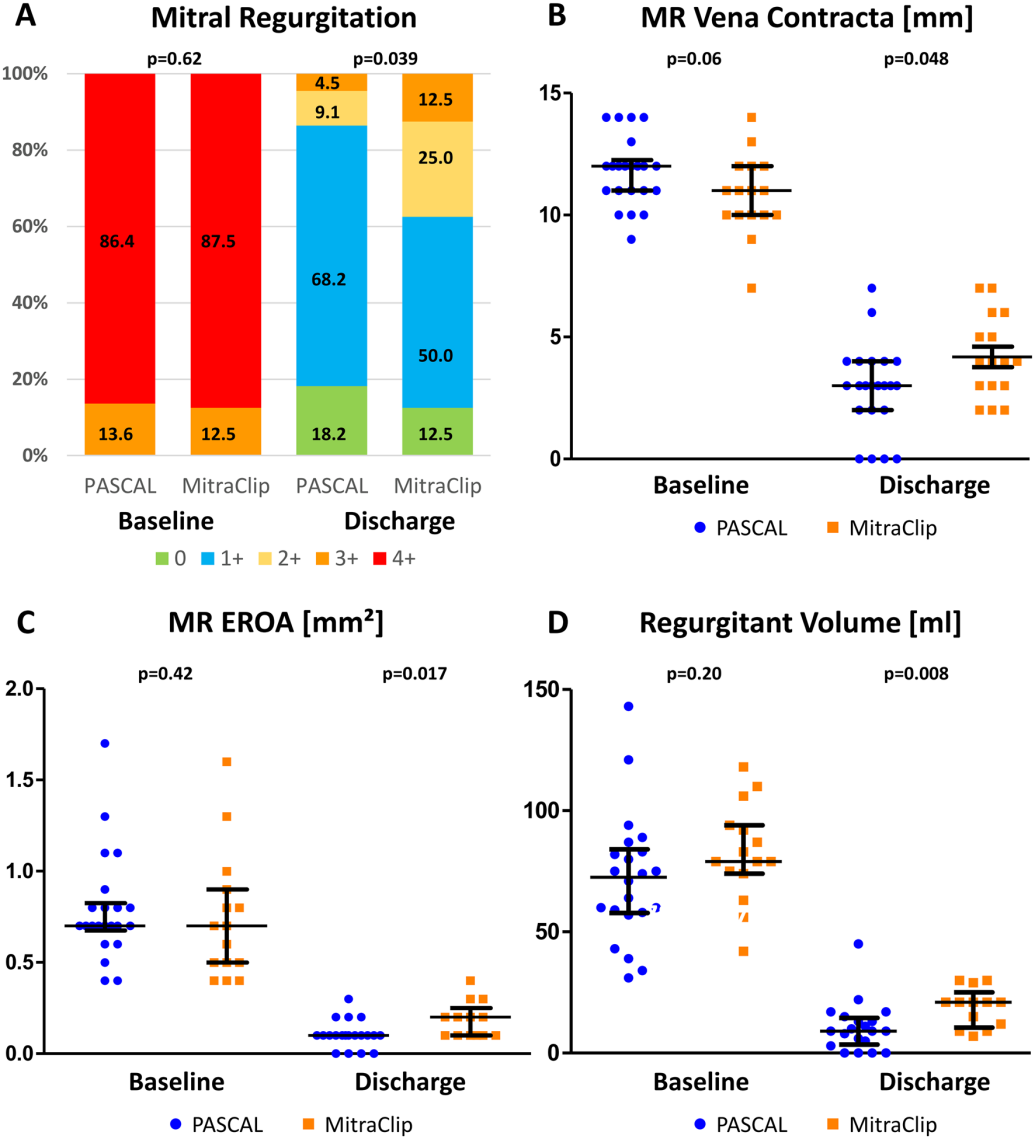
Echocardiographic parameters before and after transcatheter edge-to-edge mitral valve repair with the MitraClip and PASCAL system. Postprocedural mitral regurgitation grade (**a**), vena contracta (**b**), effective regurgitant orifice area (**c**) and regurgitant volume (**d**) were significant lower in the PASCAL group

The postprocedural echocardiography was performed before discharged, in median 2 [1–3] days (PA) and 2 [1–4] days (MC) after mitral intervention. In 86.4% of the patients in the PASCAL group, a residual MR grade ≤ 1 + was achieved (Fig. 2). In contrast, MR grade ≤ 1 + was obtained with the MitraClip system in 62.5% of cases (*p* = 0.039).

Results of the PS-matched groups are presented in Supplemental Tables 3–5. Before matching, the PS in the PASCAL and MitraCip groups were 0.47 ± 0.23 and 0.66 ± 0.17, respectively. After matching, the PS were 0.61 ± 0.16 and 0.62 ± 0.17, respectively. Periprocedural data of the matched groups confirmed an at least equivalent procedural outcome of the PASCAL device.

The transmitral gradient increased significantly in both groups (PA: *p* < 0.001; MC: *p* = 0.048) without a significant difference between the groups. Only one patient in both groups showed a mitral gradient > 6 mmHg. PAPsys did not show a significant difference between PA and MC group (*p* = 0.23) post-procedurally, but decreased significantly in both groups when compared to baseline values (*p* < 0.001). In addition, in a median follow up time of 367 [170–428] days three patients died (two in the PASCAL group and one in the MitraClip group; *p* = 0.12) and another three patients (one in the PASCAL group and two in the MitraClip group; *p* = 0.47) were hospitalized once for cardiac decompensation.

## Discussion

This study reveals that in patients with complex primary mitral regurgitation the transcatheter MitraClip and the PASCAL system achieve a very high degree of safety and procedural success defined as a reduction to MR grade ≤ 2 + . Both systems reduced qualitative and semi-quantitative parameters of MR to an at least comparable extent.

Transcatheter mitral valve repair is one of the fastest evolving fields in interventional cardiology with many different promising devices for mitral repair and replacement on the horizon. In this context, appropriate patient and device selection according to both anatomic and clinical criteria will be of paramount importance in achieving optimal outcomes. While mitral valve replacement appears as an alluring future strategy particularly for complex pathologies at first glance, its feasibility in difficult morphologies as well as its safety in true high-risk patients still have to be proven.

Against this background TMVR devices with a high therapeutic efficiency even in extensive degenerative disease of the mitral valve will be crucial to further advance the role of transcatheter mitral treatment. The cohort studied herein represents a group of patients with complex MR severity as reflected by the fact that three quarters of the studied population exhibited a flail leaflet as underlying pathology, a median EROA of 0.70 cm^2^ and a median vena contracta of 12 mm, factors which have previously been identified to be associated with an increased risk of procedural failure [10]. In comparison, in the recently presented EXPAND registry baseline EROA was 0.4 ± 0.2 cm^2^ in the patient cohort with primary MR [3].

Despite the pronounced severity of mitral valve disease in our cohort, in both the PASCAL as well as the MitraClip group a reduction of MR to grade ≤ 2 + could be achieved in over 85% of cases. Nevertheless, there is general agreement that residual MR has a prognostic impact following TMVR and that MR reduction to grade ≤ 1 + should be the intended goal [11, 12]. In this context, it is of great interest that in the present study reduction of MR to grade ≤ 1 + was achieved in 86.4% of patients with the PASCAL device and in 62.5% with MitraClip, a difference which reached statistical difference. While arguments for an advantage of the PASCAL device (e.g. larger width, separate clasp control) can be found for the studied morphologies, these findings should be interpreted with caution. Thus, the relatively small sample size of our population has to be taken into account, which carries the risk that relevant imbalances existed between both groups which remain statistically undetected. We tried to account for this by PS matching our groups and, explicitly acknowledging the limited sample size after matching, could confirm an at least equivalent procedural outcome with PASCAL (Supplemental Tables 3–5). In addition, underestimation of residual MR at postprocedural echocardiography due to more pronounced shadowing by the PASCAL device should be considered.

**Fig. 3:**
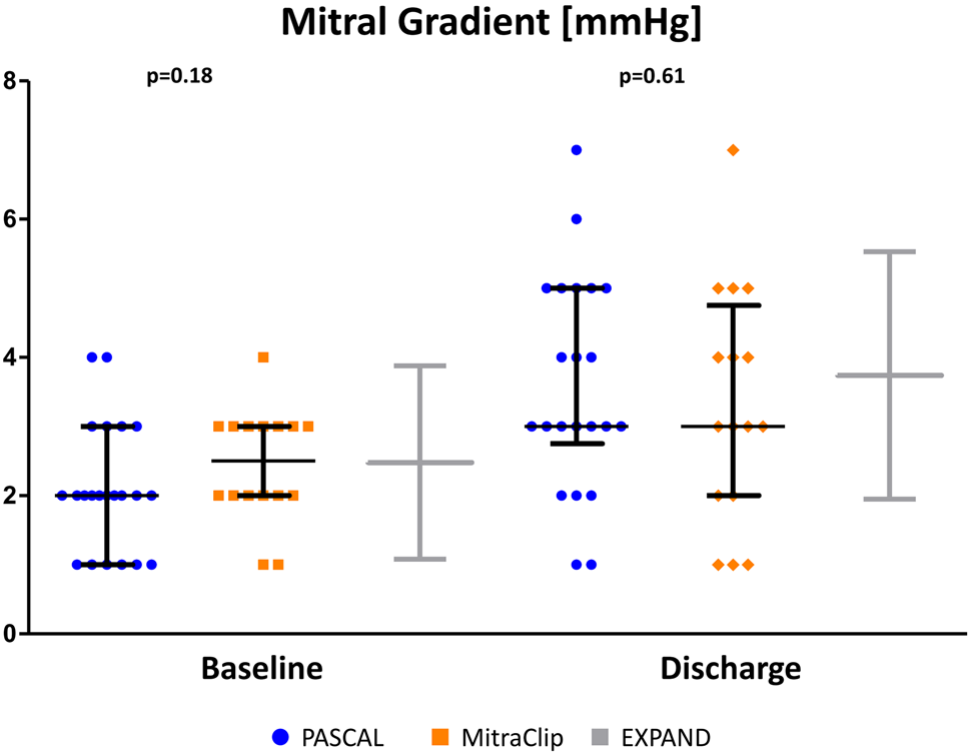
Pre- and postprocedural mitral valve gradients. Pre-procedural transmitral gradients between the groups were not different and are in line with the EXPAND registry data. The PASCAL device was not associated with an increased mitral valve gradient compared to the EXPAND registry and the MitraClip group

Regarding the postprocedural mitral gradient, our cohort is in line with the results from the EXPAND registry (Fig. 3), which showed similar postprocedural gradients in a total of 337 patients treated with MitraClip systems (XTR and NTR) [3]. Observed one-year all-cause mortality and rehospitalization rate were within the expected range [13].

### Limitations

Our study is clearly limited by the fact that sample size was relatively low, as discussed above and that our data were collected at a single center. Furthermore, device selection was not randomized but rather was performed at the discretion of the interventional cardiologist. Due to the too small sample size we were not able to test whether there was an interaction between the interventionalist and the respective device. As all physicians were adequately experienced with the MitraClip system before the start of data collection the effect of a procedural learning curve for the PASCAL device appears unlikely as it would have favored the MitraClip. However, it cannot be ruled out that in the beginning more straightforward morphologies were chosen for the PASCAL device. On the other hand, extensive echocardiographic and morphological analyses did not reveal significant differences between both groups in this regard.

The ongoing randomized controlled CLASP IID (NCT03706833) study which compares both devices in patients with primary mitral regurgitation will certainly provide deeper insights into the strengths and weaknesses of both devices. Moreover, the impact of the latest MitraClip device (Generation 4) with different clip arm widths and lengths will be of great interest.

## Conclusion

In conclusion, in the hands of experienced interventionalists our data show a robust performance of both the MitraClip and the PASCAL device in patients with complex primary mitral regurgitation.

## Supplementary Information

Below is the link to the electronic supplementary material.

Supplementary file1 (DOCX 37 kb)
