# Supplementary material for: PASCAL mitral valve repair system versus MitraClip: comparison of transcatheter edge-to-edge strategies in complex primary mitral regurgitation
Source: Clin Res Cardiol. 2021 Apr 10;110(12):1890–9. doi: 10.1007/s00392-021-01845-8 (PMC8639575; doi:10.1007/s00392-021-01845-8)
Supplement: Supplementary file 1 — Supplementary file1 (DOCX 37 kb) [file 392_2021_1845_MOESM1_ESM.docx]

Supplemental Table 1: Further Baseline Echocardiographic Parameters: Values are given as median [IQR].

| Echocardiographic Parameters | PASCAL | MitraClip | standardized difference | p-value |
| --- | --- | --- | --- | --- |
| Ejection fraction [%] | 56 [55-63] (n=22) | 61 [53-65] (n=16) | -0.30 | 0.37 |
| Left ventricular end-diastolic diameter [mm] | 57 [48-61] (n=22) | 57 [49-60] (n=16) | -0.08 | 0.83 |
| Left ventricular end-diastolic volume [ml] | 116 [72-150] (n=22) | 95 [72-154] (n=16) | 0.08 | 0.62 |
| Left ventricular end-systolic diameter [mm] | 36 [30-38] (n=22) | 37 [29-41] (n=16) | -0.09 | 0.79 |
| LV end-systolic volume [ml] | 47 [29-71] (n=22) | 39 [26-61] (n=16) | 0.20 | 0.54 |
| Left atrial volume [ml] | 118 [89-152] (n=22) | 128 [97-165] (n=16) | -0.40 | 0.43 |
| Left atrial volume index [ml] | 64 [52-89] (n=22) | 73 [55-97] (n=16) | -0.38 | 0.62 |

Supplemental Table 2: Further Parameters derived from Mitral Valve Quantification Analysis (MVQ). Values are given as median [IQR].

| MVQ Analysis | PASCAL | MitraClip | standardized difference | p-value |
| --- | --- | --- | --- | --- |
| Annulus height [mm] | 5.5 [4.4-7.1] (n=22) | 5.1 [4.1-7.5] (n=16) | 0.11 | 0.74 |
| Mitral-aortic angle [°] | 137 [123-146] (n=22) | 135 [128-146] (n=16) | -0.17 | 0.61 |
| Tenting height [cm] | 0.5 [0.2-0.9] (n=22) | 0.6 [0.5-07] (n=16) | 0.08 | 0.82 |
| Annulus area 2D [cm²] | 10.8 [9.9-12.3] (n=22) | 9.8 [7.0-14.0] (n=16) | 0.11 | 0.73 |
| Inter-trigonal distance [cm] | 2.4 [1.5-2.7] (n=22) | 2.6 [2.3-3.2] (n=16) | -0.74 | 0.08 |
| Anterior leaflet angle [°] | 12 [8-22] (n=22) | 14 [9-36] (n=16) | -0.11 | 0.58 |
| Posterior leaflet angle [°] | 11 [6-22] (n=22) | 16 [9-36] (n=16) | -0.37 | 0.29 |
| Anterior closure line length 3D [cm] | 4.1 [3.5-4.6] (n=22) | 4.1 [3.5-4.8] (n=16) | -0.28 | 0.82 |
| Posterior closure line length 3D [cm] | 4.3 [3.6-4.6] (n=22) | 4.3 [3.5-5.4] (n=16) | -0.32 | 0.75 |
| Sphericity index (mitral valve) | 0.85 [0.80-0.90] (n=22) | 0.90 [0.80-1.00] (n=16) | -0.53 | 0.11 |
| Tenting area [cm²] | 1.2 [0.7-1.9] (n=22) | 1.8 [0.7-3.2] (n=16) | -0.14 | 0.36 |
| Tenting volume [ml] | 1.5 [0.9-2.6] (n=22) | 1.2 [0.8-2.5] (n=16) | 0.16 | 0.63 |
| Tenting volume fraction [%] | 59.6 [48.6-69.9] (n=22) | 44.1 [20.9-65.9] (n=16) | 0.51 | 0.16 |
| Orifice area [cm²] | 0.2 [0.0-0.8] (n=22) | 0.6 [0.0-1.0] (n=16) | -0.18 | 0.38 |

Supplemental Table 3: Baseline Characteristics of PS matched groups. Values are given as median [IQR] or percentages (n).

| Characteristics | PASCAL (n=10) | MitraClip (n=10) | standardized difference | p-value |
| --- | --- | --- | --- | --- |
| Age | 82.0 [75.0-86.3] | 81.8 [79.5-87.0] | -0.06 | 0.68 |
| Female | 60% (6) | 70% (7) | -0.20 | 0.64 |
| Body mass index kg/m² | 27.7 [21.4-33.9] | 24.0 [22.8-30.1] | 0.47 | 0.63 |
| STS-Score (%) | 2.3 [1.8-5.4] | 2.7 [1.7-4.0] | 0.41 | 0.77 |
| EuroScore II (%) | 3.9 [2.3-7.5] | 4.4 [3.1-6.9] | -0.17 | 0.74 |
| Atrial fibrillation | 70% (7) | 60% (6) | 0.21 | 0.64 |
| Diabetes mellitus | 20% (2) | 10% (1) | 0.27 | 0.53 |
| Chronic obstructive pulmonary disease | 20% (2) | 20% (2) | 0.00 | >0.99 |
| Coronary artery disease | 30% (3) | 40% (4) | -0.21 | 0.64 |
| History of myocardial infarction | 0% (0) | 10% (1) | -0.47 | 0.53 |
| History of cardiac surgery | 10% (1) | 10% (1) | 0.00 | >0.99 |
| Extracardiac arteriopathy | 10% (1) | 20% (2) | 0.00 | >0.99 |
| Stroke | 20% (2) | 20% (2) | -0.27 | 0.53 |
| Dialysis | 0% (0) | 0% (0) | 0.00 | 0.14 |
| ICD/CRT-Device | 20% (2) | 0% (0) | -0.67 | 0.28 |
| NTpro-BNP [pg/ml] | 2981 [522-4885] (n=10) | 3000 [379-3715] (n=9) | 0.32 | 0.85 |

Supplemental Table 4: Baseline Echocardiographic Parameters of the PS matched groups: Values are given as median [IQR] or percentages (n).

| Echocardiographic Parameters | PASCAL (n=10) | MitraClip (n=10) | standardized difference | p-  value |
| --- | --- | --- | --- | --- |
| Mitral regurgitation degree [I-IV] | **III:** 20% (2)  **IV:** 80% (8)  IOR 0.776 | **III:** 10% (2)  **IV:** 90 % (14)  IOR 0.636 | -0.03 | 0.62 |
| MV pathology | **Prolapse:** 30% (3)  **Flail:** 70% (7) | **Prolapse:** 20% (2)  **Flail:** 80% (8) | -0.02 | 0.88 |
| Vena contracta [mm] | 11 [10-12] | 11 [10-12] | -0.81 | 0.53 |
| Effective regurgitant orifice area [cm²] | 0.70 [0.55-0.73] | 0.70 [0.50-0.90] | -0.03 | >0.99 |
| Regurgitant volume [ml] | 78 [38-96] | 83 [74-93] | -0.30 | 0.45 |
| Proximal isovelocity surface area (PISA) radius adjusted to Nyquist limit 30–40 cm/s [mm] baseline | 11 [10-12] | 11 [10-13] | -0.26 | 0.28 |
| Flail gap [mm] | 2.5 [1.5-6] (n=7) | 2.0 [0-7] (n=8) | -0.61 | 0.28 |
| Flail width [mm] | 9.0 [7.0-10.0] (n=7) | 8.5 [7.3-11.0] (n=8) | -0.31 | 0.61 |
| Transmitral antegrade gradient [mmHg] | 2.0 [1.8-3.0] | 3.0 [2.0-3.0] | -0.84 | 0.12 |
| Mitral valve orifice area [cm²] | 4.5 [4.1-5.9] | 4.2 [3.8-4.7] | -0.47 | 0.25 |
| Annulus area 3D [cm²] | 12.0 [10.7-13.2] | 10.4 [8.4-13.2] | -0.43 | 0.22 |
| Annulus perimeter [cm] | 12.5 [12.1-13.5] | 11.7 [10.5-13.0] | 0.50 | 0.22 |
| A-P diameter [cm] | 3.4 [3.0-3.8] | 3.1 [3.0-3.6] | 0.23 | 0.48 |
| AL-PM diameter [cm] | 3.9 [3.7-4.1]) | 3.5 [3.1-4.0] | 0.91 | 0.89 |
| Anterior leaflet length [cm] | 2.1 [1.9-2.2] | 2.2 [1.9-2.6] | -0.43 | 0.44 |
| Posterior leaflet length [cm] | 1.7 [1.3-2.2] | 1.4 [1.3-2.4] | 0.09 | 0.68 |
| Anterior leaflet area [cm²] | 5.4 [5.2-6.3] | 5.4 [4.1-5.9] | -0.49 | 0.44 |
| Posterior leaflet area [cm²] | 7.6 [5.9-8.7]) | 7.1 [4.1-9.6] | -0.13 | 0.97 |
| Commissural diameter [cm] | 3.7 [3.6-4.2] | 3.2 [3.0-3.8] | 0.12 | 0.06 |
| Tricuspid regurgitation  [0-V] | **0:** 10% (1)  **I:** 60 % (6)  **II:** 30 % (1) | **I:** 60 % (6)  **II:** 40 % (4) | -0.31 | 0.31 |
| Estimated systolic pulmonary arterial pressure [mmHg] | 38 [28-56] | 48 [36-69] | -0.45 | 0.44 |

Supplemental Table 5: Peri- and Postprocedural Results of Transcatheter Mitral Valve Repair with the PASCAL and MitraClip System in PS matched groups. Values are given as median [IQR] or percentages (n).

| Peri- and Postprocedural Parameters | PASCAL (n=10) | MitraClip (n=10) | p-value |
| --- | --- | --- | --- |
| Number of implanted devices | 2 [1-2] | 2 [1-2] | >0.99 |
| Procedure time [min] | 97.5 [81.3-122.3] | 91.5 [70.5-116.3] | 0.28 |
| Fluoroscopy time [min] | 8.7 [5.8-11.9] | 8.8 [6.6-11.6] | 0.85 |
| Radiation dose area product [cGy*cm²] | 368.1 [194.6-681.8] | 290.1 [201.8-436.4] | 0.53 |
| Mitral regurgitation degree [0-IV] | **0:** 20% (2)  **I:** 70% (15)  **II:** 10% (2) | **0:** 0%  **I:** 50% (5)  **II:** 40% (4)  **III:** 10% (1) | 0.16 |
| Effective regurgitant orifice area [cm²] | 0.11 [0.10-0.15] | 0.15 [0.10-0.20] | 0.24 |
| Regurgitant volume [ml] | 11 [4-17] | 21 [11-21] | 0.11 |
| Vena contracta [mm] | 3 [3-3] | 4 [3-5] | **0.043** |
| PISA radius adjusted to Nyquist limit 30–40 cm/s [mm] | 3 [3-4] | 4 [3-5] | 0.28 |
| Transmitral gradient [mmHg] | 3.7 [2.0-5.0] | 4.0 [2.8-8.0] | 0.48 |
| Estimated systolic pulmonary arterial pressure [mmHg] | 33 [24-38] | 32 [23-35] | 0.84 |
| Δ Vena contracta (Baseline-Discharge) [mm] | 9 [7-10] | 7 [6-8] | **0.043** |
| Δ Effective regurgitant orifice area (Baseline-Discharge) [cm²] | 0.60 [0.35-0.60] | 0.50 [0.30-0.70] | 0.92 |
| Δ Regurgitant Volume (Baseline-Discharge) [ml] | 58 [26-77] | 68 [53-79] | 0.30 |
| Δ Pisa radius adjusted to Nyquist limit 30–40 cm/s (Baseline-Discharge) [mm] | 7 [6-10] | 7 [6-8] | 0.74 |
